# Prognostic value of the SYNTAX score on myocardial injury and salvage in STEMI patients after primary percutaneous coronary intervention: a single-center retrospective observational study

**DOI:** 10.1186/s12872-021-02395-7

**Published:** 2021-12-09

**Authors:** Guangren Gao, Lianrong Feng, Jinguo Fu, Yi Li, Zhaoyang Huo, Lei Zhang, Lei Wang, Heping Niu, Liqing Kang, Jun Zhang

**Affiliations:** 1 Department of Cardiology, Cangzhou Central Hospital, Tianjin Medical University, Tianjin, China; 2 Department of Neurology, Cangzhou Central Hospital, Tianjin Medical University, Tianjin, China; 3 Department of Cardiology, General Hospital of Northern Theater Command, Shenyang, Liaoning China; 4 Department of Magnetic Resonance Imaging, Cangzhou Central Hospital, Tianjin Medical University, Tianjin, China

**Keywords:** ST-elevation myocardial infarction, Percutaneous coronary intervention, Coronary vessels, Cardiac magnetic resonance, Prognosis, SYNTAX score

## Abstract

**Background:**

SYNTAX score (SS) was shown to positively correlate with postprocedural myocardial injury in patients after elective coronary artery intervention, but evidence about the association of SS with myocardial salvage in ST-segment elevation myocardial infarction (STEMI) patients is still needed. This study aimed to evaluate the prognostic value of SS for myocardial injury and salvage assessed by cardiac magnetic resonance (CMR) after primary percutaneous coronary intervention (PCI) in STEMI patients.

**Methods:**

This single-center retrospective study consecutively enrolled STEMI patients who had undergone primary PCI within 12 h from symptom onset. Both angiography and CMR were performed during hospitalization, and patients were divided into low SS (SS ≤ 22), mediate-high SS (SS > 22) groups. Correlation and multivariable analyses were performed.

**Results:**

A total of 149 STEMI patients (96 low SS, 53 mediate-high SS) were included. In terms of myocardial injury parameters, there was a positive correlation (*p* < 0.001, Spearman r = 0.292) between SS and infarct size (IS), and a negative correlation (*p* < 0.001, Spearman r =  − 0.314) between SS and myocardial salvage index (MSI). In the multivariable model, SS (> 22 as categorical variable, OR = 2.245, 95% CI [1.002–5.053], *p* = 0.048; as continuous variable, OR = 1.053, 95% CI [1.014–1.095], *p* = 0.008) was significantly associated with high IS (≥ mean 35.43). The areas under the receiver operating characteristic (ROC) curves of SS for high IS and low MSI (≤ median 28.01) were 0.664 and 0.610.

**Conclusions:**

In STEMI patients undergoing primary PCI, SYNTAX score positively correlated with infarct size and negatively with myocardial salvage, indicating an independent predictive value of the myocardial injury.

## Background

Recovery from reperfused myocardial infarction (MI) is burdened by the occurrence of dynamic tissue changes, such as edema, inflammation, microvascular obstruction and hemorrhage [1, 2]. Microvascular obstruction (MVO) is the underlying cause for the no-reflow phenomenon in ST-segment elevation myocardial infarction (STEMI); it is strongly associated with mortality and hospitalization for heart failure (HF) within 1 year; and is considered to be a main secondary endpoint with the independent predictive value of long-term major cardiovascular adverse events (MACE) [2–4]. Furthermore, in acute MI patients, a high extent of myocardial loss after STEMI (infarct size, IS) and intramyocardial hemorrhage (IMH) is closely related to all-cause mortality and hospitalization for HF or MACE [5, 6]. Accordingly, myocardial salvage, defined as the amount of myocardium that is jeopardized by a coronary occlusion but spared from infarction, can be used to compare different treatment options so that strategies that benefit salvage could be implemented in clinical practice [7–10].

Early assessment of myocardial salvage is the most important during the intense care phase. Cardiac magnetic resonance (CMR) is the gold-standard technique for noninvasive myocardial tissue characterization. It is increasingly used for improved long-term risk stratification of post-MI patients or initial evaluation preceding percutaneous coronary intervention (PCI) [2]. Typical indexes of CMR measured 3 to 7 days post-MI are recommended as CMR endpoints in clinical trials [11, 12]. Still, a more integrated approach is often needed to evaluate the complex coronary artery disease (CAD) and the prognosis of STEMI.

The anatomical Synergy between PCI with Taxus and Cardiac Surgery (SYNTAX score, SS) is an important angiographic scoring system that can help clinicians establish the optimum revascularization approach in patients with CAD [13–16]. Previous studies proved that SS was an independent predictor of MACE and 3 year-mortality after primary PCI [17, 18]. It was also found that SS correlated significantly with cardiac troponin releases after elective PCI and could predict peri-procedural myocardial injury defined as elevated troponin I at 6–24 h post-PCI [19, 20]. However, studies on the association of SS with myocardial injury and salvage as CMR endpoints are rare, and mostly limited to case reports.

Based on the above, the study aimed to evaluate the prognostic value of SS for myocardial injury and salvage assessed by CMR in STEMI patients after primary PCI.

## Methods

### Study design and participants

This single-center retrospective observational study enrolled STEMI patients who underwent primary PCI at Cangzhou central hospital (tertiary care hospital) between October 2018 and September 2020. Inclusion criteria were as follows: (1) electrocardiography (ECG) features consistent with acute STEMI in accordance to Fourth Universal Definition of Myocardial Infarction [1]; (2) time duration within 12 h from typical chest pain to primary PCI; (3) CMR within 3–10 days from symptom onset. In addition, patients were excluded for the following reasons: (1) old myocardial infarction history; (2) poor image quality; (3) declined CMR for personal reasons. The present study was approved by the ethical committee of Cangzhou Central Hospital (2020-286-01). The requirement for informed consent was waived by the ethical committee of Cangzhou Central Hospital due to the retrospective nature of the study.

### PCI procedures

Primary PCI was performed based on current guidelines [21]. Patients were administered a loading dosage of aspirin 300 mg (Bayer, 100 mg) and clopidogrel 300 mg (Plavix, 75 mg) or ticagrelor 180 mg (BRILINTA, 90 mg) before coronary angiography and PCI was performed via radial access using a 6-F arterial sheath. After access was established, 2500 U of unfractionated heparin was injected through the sheath. Coronary angiography was performed using a 6-F IL 3.5 multifunctional guiding catheter (Terumo, Tokyo, Japan). ECG and invasive blood pressure monitoring were continuously performed during the surgery. For PCI, additional heparin (total 100 U/kg, including the previous dose) was routinely administered through the arterial sheath. The guiding catheter was deployed at the ostium of the coronary artery for diagnostic angiography. A revascularization strategy (balloon angioplasty only or stenting) was chosen based on the surgeon’s decision. After the procedure, patients were administered a maintenance dosage of aspirin 100 mg daily and ticagrelor 90 mg twice a day or clopidogrel 75 mg once a day. Additional medications, such as statins, β-receptor blockers, angiotensin-converting enzyme inhibitors/angiotensin receptor blockers, glycoprotein IIb/IIIa inhibitors, and low-molecular-weight heparin, were administered based on the recommended guidelines unless contraindicated. Pre- and post-PCI antegrade flow in the infarct-related artery was characterized using the TIMI system.

### SYNTAX score

The SS for each patient was calculated retrospectively, based on scoring for all coronary lesions with diameter stenosis > 50%, and vessels with diameter > 1.5 mm, using the SS algorithm, as recommended [14, 16]. The calculation was done using an openly accessible web-based score calculator (http://www.syntaxscore.com). Based on the previous reports [22, 23] SS was categorized as low (≤ 22), intermediate (22 < SYNTAX score ≤ 32), or high (≥ 33), and patients were divided into low SS and mediate-high SS groups. The SYNTAX score was assessed visually by two experienced interventional cardiologists trained to perform SYNTAX score assessments and blinded to the treatment and CMR data. Every significant inter-observer difference required a recalculation.

### Laboratory tests, echocardiography and CMR

Blood samples were taken immediately after hospitalization, and echocardiography was performed on 5 (3–7) days from symptom onset. CMR was performed on 7.15 (5.26–8.24) days from symptom onset using a 3.0-T scanner (GE Discovery MR750w; GE Healthcare, Milwaukee, WI, USA) with electrocardiographic-gated image acquisition, as described in previous studies [24, 25]. MRI parameters were measured on short-axis images covering the entire left ventricle (8-/0-mm slice thickness/slice gap) with the following sequences: a steady-state free precession (SSFP) cine sequence to determine the left ventricular (LV) function, mass and volume, and a short-tau inversion recovery T2-weighted (T2-STIR) sequence to determine the area at risk (AAR) of myocardial infarction. Late gadolinium enhancement (LGE) images were acquired approximately 10 ~ 15 min after the intravenous administration of gadolinium-based contrast medium (0.2 mmol/kg, Magnevist, gadopentetate dimeglumine injection, Bayer) to determine the IS.

Analysis was performed using dedicated software (cmr42 version 5.11.3, Circle Cardiovascular Imaging, Calgary, Alberta, Canada). Images were anonymized, batched, and analyzed in a blinded fashion by two experienced operators. The AAR was defined as a high-signal myocardial edema mass/LV mass ratio. The IS was defined as the hyperenhanced myocardium on the LGE images and is expressed as the infarcted LV mass/LV mass ratio. MVO was defined as dark areas surrounded by hyperenhanced myocardium on the LGE images. The presence of IMH was defined as hypointense areas within the brighter edematous zone on T2-STIR images. The papillary muscles were included in the LV cavity volume. The regions of interest for the volumes of AAR, IMH, IS, and MVO were created by manually drawing the lesion contours. In contrast, the LV volume was calculated by the semiautomated drawing of endocardial and epicardial contours on each slice for the whole LV myocardium. Myocardial edema was described as areas with a signal intensity > 5 standard deviations (SD) that of remote normal myocardium. The IS was calculated using the > 5 SD method. Discordant cases were reviewed and reconciled with superior imaging specialists. The myocardial salvage index (MSI) was defined as (AAR − IS)/AAR × 100.

For echocardiographic evaluation, Xcelera R4.1 (Philips Medical Systems) was used. Echocardiographic morphological and functional parameters were measured according to the definitions and recommendations from the available American Society of Echocardiography guidelines [26].

### Statistical analysis

Categorical data are presented as numbers and percentages, and continuous data are presented as the mean (standard deviation) or median (interquartile range). The Shapiro–Wilk test was applied to assess for data normality. Continuous variables with normal distributions were compared using the t-test. Continuous parameters that were not normally distributed were compared using the Mann–Whitney test. Categorical variables were compared using the chi-squared test (Fisher’s exact test when the expected value was < 5). Spearman’s correlation coefficient was used to analyze associations between SS and CMR or echocardiographic parameters. Independent predictors of high IS and low MSI were determined in a multivariable binary logistic regression model using the backward stepwise method adjusted for all baseline variables found significant (*p* < 0.05) in the univariable logistic regression model. Receiver operating characteristic (ROC) curves were generated to determine the usefulness of SS to discriminate high IS and low MSI. All statistical analyses were performed using SPSS version 20 (IBM, Armonk, NY, USA) and graphs were generated using GraphPad Prism version 8.0 (GraphPad Software, La Jolla, CA, USA). *P*-value of < 0.05 was regarded as statistically significant.

## Results

### Baseline characteristics of patients

**Fig. 1:**
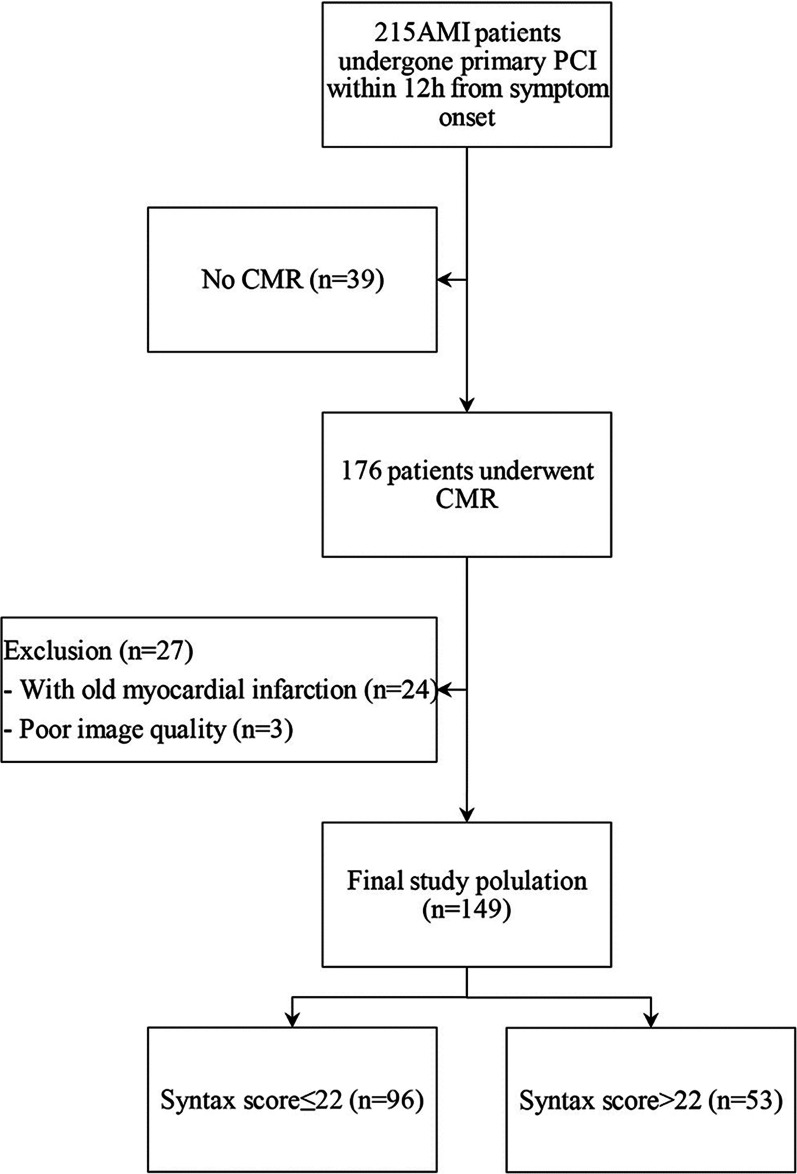
Study flow chart of patient enrollment. *AMI* acute myocardial infarction; *PCI* percutaneous coronary intervention; *CMR* cardiac magnetic resonance imaging

**Fig. 2:**
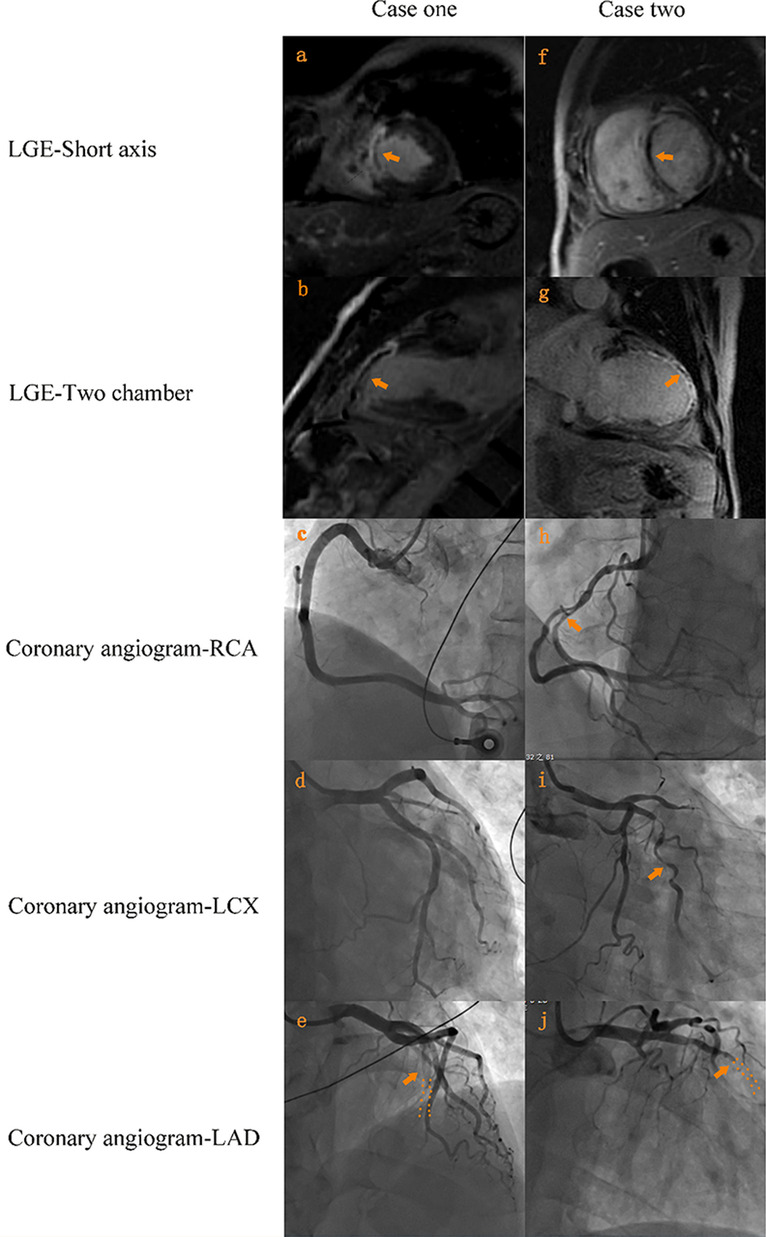
Two case examples of arteriography and cardiac magnetic resonance image. Case one (**a**–**e**): A 59 years old man with anterior ST-elevation myocardial infarction was identified to have a single total occluded LAD (SYNTAX score 18.5 and infarct size 22.74); Case two (**f**–**j**): A 64 years old man also with anterior ST-elevation myocardial infarction was identified to have total occluded LAD and diffused diseased intermediate branch and RCA (SYNTAX score 32.5 and infarct size 54.91). *LAD* left anterior descending artery; *RCA* right coronary artery

A total of 215 AMI patients who underwent primary PCI within 12 h from symptom onset were retrospectively included. Out of the 39 with no CMR data, 24 with old myocardial infarction, and three with poor image quality were excluded. Finally, 149 patients (mean age 59.89 ± 10.86 years, 72.5% male) were enrolled for analysis (Fig. 1 and Table 1). Examples of obtained arteriographic results are illustrated in Fig. 2. According to SS (17[9–25]), patients were divided into low SS (≤ 22, n = 96) and mediate-high SS (> 22, n = 53) groups. Differences in baseline characteristics between the two groups are shown in Table 1. Compared with the low SS group, patients in the mediate-high SS group were older (58.32 ± 11.47 vs. 62.72 ± 9.09, *p* = 0.011), and had a lower BMI (26.25 ± 3.62 vs. 24.92 ± 2.86, *p* = 0.023). The prevalence of multivessel disease (*p* < 0.001), initial TIMI thrombus grade 4/5 (*p* = 0.002), initial TIMI flow grade 0/1 (*p* = 0.008), and APOB (*p* = 0.038) were higher but creatinine clearance rate (*p* = 0.016) was lower in the mediate-high SS group. There were no differences between groups concerning medical history, culprit lesion, mode of reperfusion, and medications after surgery (all *p* > 0.05).

**Table 1:** Demographic and Clinical characteristics of STEMI patients grouped by SYNTAX score

Characteristics	Whole (n = 149)	SS ≤ 22 (n = 96)	SS > 22 (n = 53)	*p*-value
Age (years)	59.89 ± 10.86	58.32 ± 11.47	62.72 ± 9.09	0.011
Male	108 (72.5)	68 (70.8)	40 (75.5)	0.572
BMI (kg/m^2^)	25.78 ± 3.42	26.25 ± 3.62	24.92 ± 2.86	0.023
Hypertension	64 (43.0)	45 (46.9)	19 (35.8)	0.228
Smoking	91 (61.1)	56 (58.3)	35 (66.0)	0.385
Hyperlipidemia	17 (11.4)	11 (11.5)	6 (11.3)	1.0
Diabetes	35 (23.5)	21 (21.9)	14 (26.4)	0.550
Initial heart rate	76.79 ± 13.35	76.53 ± 11.69	77.27 ± 16.07	0.645
GRACE score	109.97 ± 22.73	107.32 ± 22.81	114.75 ± 22.06	0.089
CRUSADE score	18.0 (9.0,24.0)	15.0 (9.0,22.0)	18.5 (12.8,29.3)	0.056
Procedural data for the patients				
Time of pain to door				
≤ 6 h	99 (66.4)	60 (62.5)	39 (73.6)	0.206
> 6 h	50 (33.6)	36 (37.5)	14 (26.4)	
Time of door to reperfusion/angiography only (h)	1.17 (0.68,80.91)	1.23 (0.69,10.10)	0.98 (0.63,234.73)	0.830
Time of pain to reperfusion/ angiography only (h)	7.05 (4.44,95.91)	7.33 (4.68,23.39)	6.22 (4.27,246.73)	0.629
Culprit lesion				
LAD	60 (40.3)	37 (38.5)	23 (43.4)	0.603
Non-LAD	89 (59.7)	59 (61.5)	30 (56.6)	
No. of diseased vessels				
0	4 (2.7)	4 (4.2)	0 (0)	< 0.001
1	54 (36.2)	51 (53.1)	3 (5.7)	
2	45 (30.2)	29 (30.2)	16 (30.2)	
3	46 (30.9)	12 (12.5)	34 (64.2)	
Initial TIMI flow grade				
0–1	93 (62.4)	52 (54.2)	41 (77.4)	0.008
2–3	56 (37.6)	44 (45.8)	12 (22.6)	
Initial TIMI thrombus grade				
0–3	56 (37.6)	45 (46.9)	11 (20.8)	0.002
4–5	93 (62.4)	51 (53.1)	42 (79.2)	
Final TIMI flow grade				
0/1	9 (6.0)	5 (5.2)	4 (7.5)	0.721
2/3	140 (94.0)	91 (94.8)	49 (92.5)	
Post-dilatation in patients with stent implanting				
Yes	86 (81.9)	57 (81.4)	29 (82.9)	0.858
No	19 (18.1)	13 (18.6)	6 (17.1)	
Mode of reperfusion (n, %)				
Balloon angioplasty	29 (19.5)	17 (17.7)	12 (22.6)	0.675
PCI with stent	105 (70.5)	70 (72.9)	35 (66.0)	
Angiography only	15 (10.1)	9 (9.4)	6 (11.3)	
Laboratory data				
Creatinine clearance rate ( mL/min)	102.24 (77.99,123.64)	111.12 (79.81,127.97)	92.11 (72.60,108.06)	0.016
cTnI (ng/ml)	0.32 (0.03,4.08)	0.35 (0.06,5.81)	0.18 (0.03,2.27)	0.207
WBC (10^9^/L)	10.51 ± 3.62	10.64 ± 3.69	10.27 ± 3.53	0.566
AST (U/L)	29.35 (19.03,61.88)	31.70 (19.85,67.0)	26.80 (18.60,55.20)	0.378
CK (U/L)	176.0 (108.5,579.0)	218.0 (110.75,616.0)	158.0 (96.0,558.25)	0.337
CKMB (U/L)	25.55 (16.13,75.90)	25.85 (16.33,61.68)	24.5 (16.0,83.90)	0.821
LDL ( mmol/L)	2.96 ± 0.85	2.89 ± 0.76	3.07 ± 0.99	0.272
APOA (g/L)	1.06 ± 0.22	1.06 ± 0.22	1.05 ± 0.21	0.704
APOB (g/L)	0.99 ± 0.26	0.96 ± 0.22	1.06 ± 0.30	0.038
Medication				
Aspirin	146 (98.0%)	93 (96.9)	53 (100.0)	0.553
Clopidogrel	46 (30.9)	33 (34.4)	13 (24.5)	0.358
Ticagrelor	101(67.8)	62(64.6)	39(73.6)	
No clopidogrel or ticagrelor	2(1.3)	1(1.0)	1(1.9)	
Statin	143(96.0)	92(95.8)	51(96.2)	1.0
Beta blocker	86(57.7)	56(58.3)	30(56.6)	0.864
ACEI	6(4.0)	3(3.1)	3(5.7)	0.666
ARB	29(19.5)	17(17.7)	12(22.6)	0.519

Categorical data are presented as absolute (percentage), normally distributed data as mean ± standard deviation, and other continuous data with medians including first and third quartiles in brackets

*BMI* body weight index; *GRACE* the Global Registry of Acute Coronary Events; *CRUSADE* Can Rapid Risk Stratification of Unstable Angina Patients Suppress Adverse Outcomes With Early Implementation of the American College of Cardiology/American Heart Association Guidelines; *LAD* left anterior descending artery; *TIMI* thrombolysis in myocardial infarction; *WBC* white blood cell; *LDL* low-density lipoprotein; *ACEI* Angiotensin-Converting Enzyme Inhibitors; *ARB* Angiotensin Receptor Blocker

### Transthoracic echocardiography and CMR parameters

**Fig. 3:**
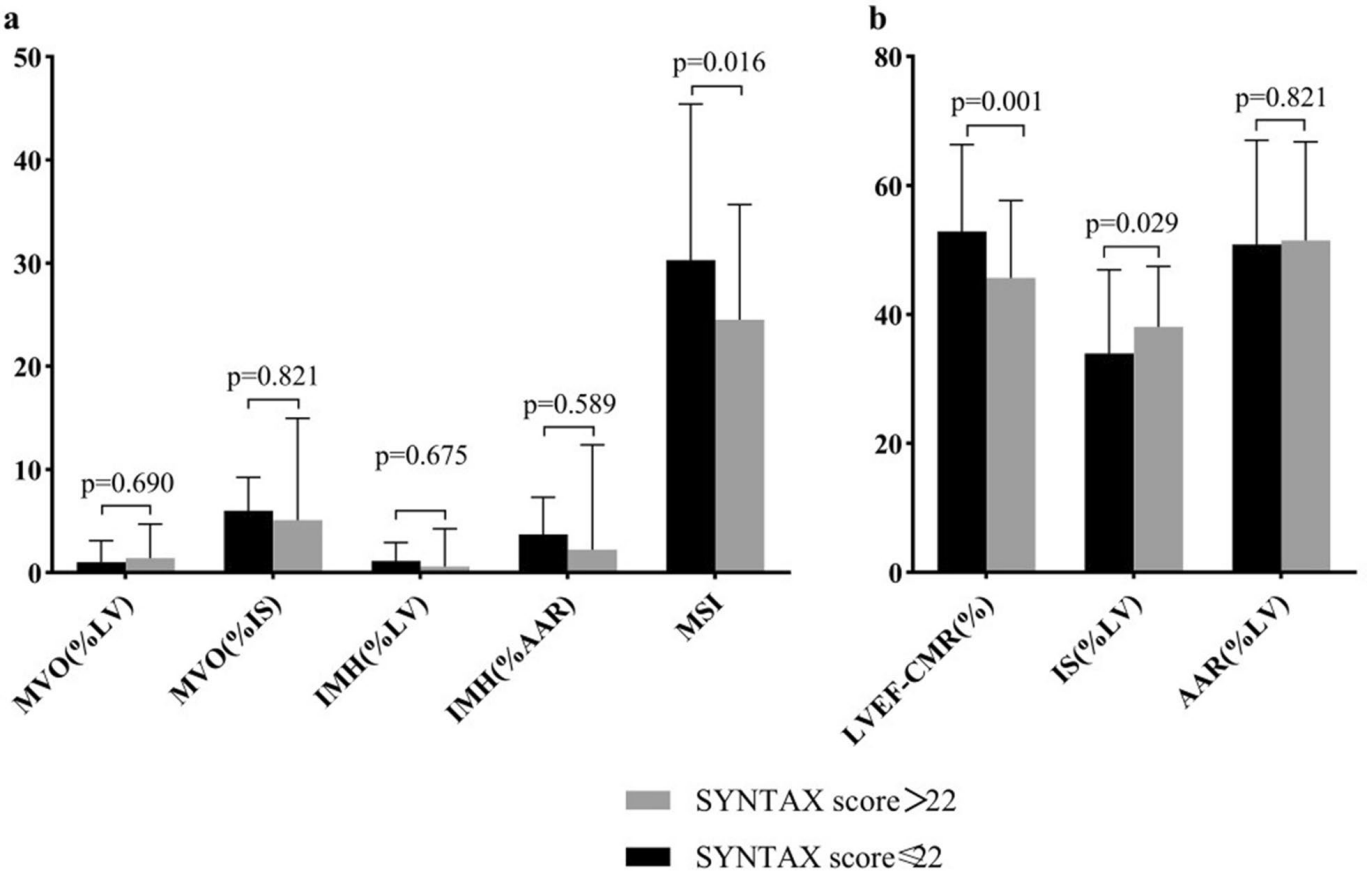
Comparison of CMR parameters between the mediate-high SS group and low SS group (**a**: non-normal distributed parameters; **b**: normal distributed parameters). *MVO* microvascular obstruction; *IMH* intramyocardial hemorrhage; *LVEF* left ventricular ejection fraction; *CMR* cardiac magnetic resonance imaging; *IS* infarct size; *LV* left ventricular; *AAR* area at risk; *MSI* myocardial salvage index

All transthoracic echocardiography (TTE) parameters were comparable between the two groups (Table 2). Whereas CMR showed a lower left ventricular ejection fraction (LVEF, 52.86 ± 13.45 vs. 45.65 ± 12.01%, *p* = 0.001), MSI (30.30[19.23–45.41] vs. 24.52[13.92–35.69] %, *p* = 0.016) and higher left ventricular end-systolic volume (LVESV, 51.28[38.23,72.17] vs. 63.96[51.34–80.38] mL, *p* = 0.029), and IS (33.93 ± 11.94 vs. 38.06 ± 9.40%LV, *p* = 0.029) in mediate-high SS group patients when compared with those in low SS group. There was no significant difference between the two groups in terms of MVO, IMH and AAR (Table 2 and Fig. 3).

**Table 2:** Transthoracic echocardiography and CMR imaging parameters stratified according to SYNTAX score

Parameters	Whole (n = 149)	SS ≤ 22 (n = 96)	SS > 22 (n = 53)	*p*-value
TTE				
LVEF (%)	56.60 ± 9.19	57.63 ± 8.87	54.77 ± 9.55	0.070
LVEDD (mm)	49.32 ± 6.30	49.33 ± 5.43	49.29 ± 7.67	0.970
LAD (mm)	37.75 ± 4.96	37.55 ± 4.68	38.12 ± 5.48	0.512
CMR				
Symptom to CMR (days)	7.31 ± 2.60	7.22 ± 2.77	7.46 ± 2.27	0.589
LVEF (%)	50.29 ± 13.37	52.86 ± 13.45	45.65 ± 12.01	0.001
LVEDV (mL)	119.12(101.19,141.06)	117.31(102.89,136.79)	125.40(93.88,148.14)	0.262
LVESV (mL)	55.01(43.99,75.05)	51.28(38.23,72.17)	63.96(51.34,80.38)	0.004
IS (% LV)	35.43 ± 11.94	33.97 ± 12.95	38.06 ± 9.40	0.029
AAR (% LV)	51.05 ± 15.83	50.83 ± 16.18	51.46 ± 15.32	0.821
Number of MVO	54(36.2)	35(36.5)	19(35.8)	1.0
MVO (mL)	1.93(0.48,4.74)	1.72(0.45,4.55)	2.22(0.49,6.44)	0.765
MVO (%LV)	1.35(0.40,3.20)	1.0(0.4,3.1)	1.4(0.4,4.7)	0.690
MVO (%IS)	5.94(1.54,9.27)	6.01(1.48,9.25)	5.10(1.62,14.95)	0.821
Number of IMH	32(21.5)	22(22.9)	10(18.9)	0.678
IMH (mL)	1.46(0.56,5.01)	1.75(0.68,4.88)	1.08(0.42,6.35)	0.535
IMH (%LV)	0.87(0.48,2.97)	1.13(0.47,2.93)	0.59(0.40,4.24)	0.675
IMH (%AAR)	3.25(1.10,8.18)	3.70(1.62,7.31)	2.21(0.79,12.39)	0.589
MSI (%)	28.01(15.94,41.81)	30.30(19.23,45.41)	24.52(13.92,35.69)	0.016

Categorical data are presented as absolute (percentage), data as mean ± standard deviation, and other continuous data with medians including first and third quartiles in brackets

*TTE* transthoracic echocardiography; *CMR* cardiac magnetic resonance imaging; *LVEF* left ventricular ejection fraction; *LVEDV* left ventricular end-diastolic volume; *LVESV* left ventricular end-systolic volume; *IS* infarct size; *LV* left ventricular; *MVO* microvascular obstruction; *AAR* area at risk; *IMH* intramyocardial hemorrhage; *MSI* myocardial salvage index

### Correlations between baseline variables and CMR parameters

**Fig. 4:**
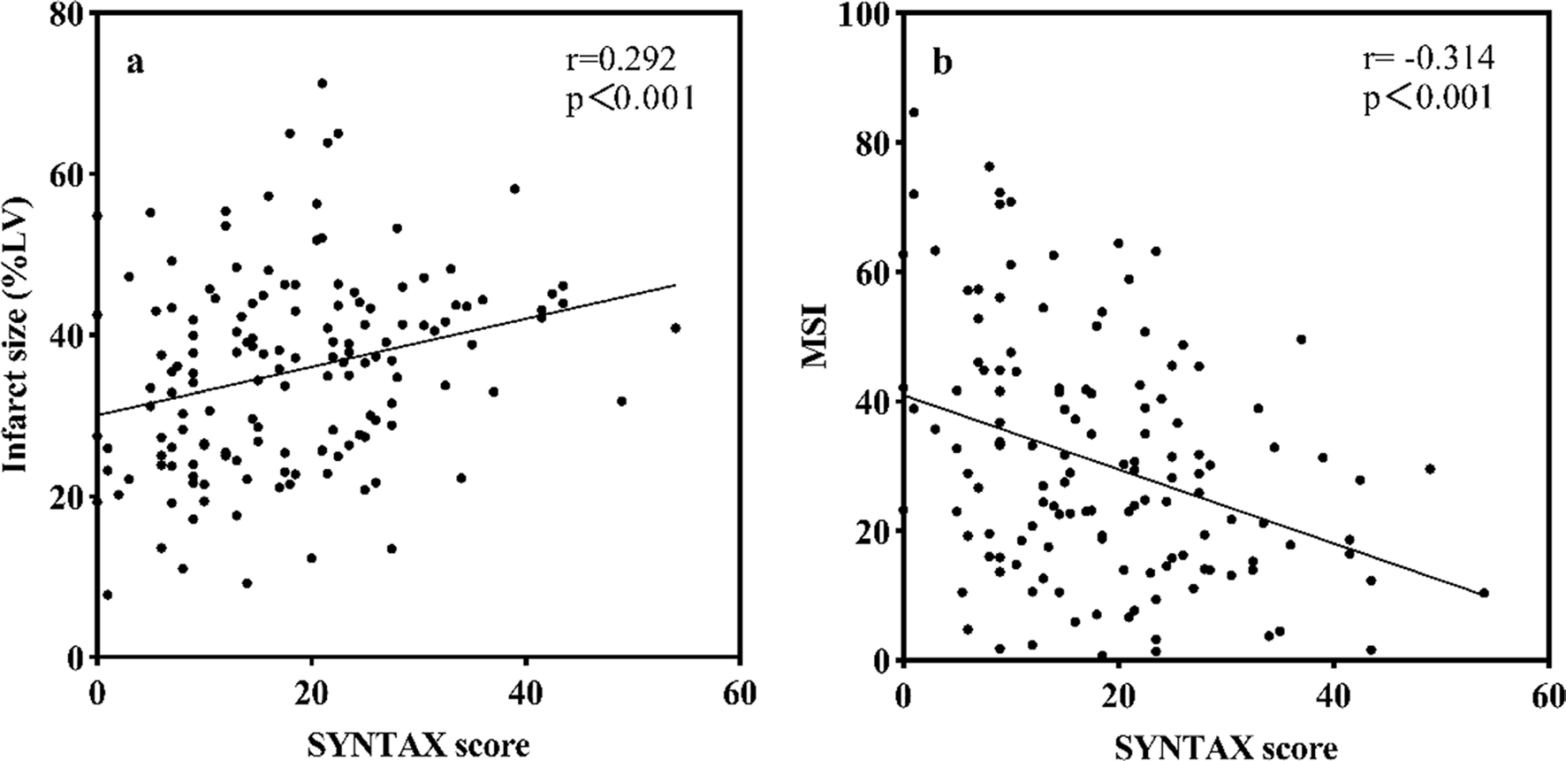
Correlations of SYNTAX score with infarct size (%LV) and MSI. *LV* left ventricular; *MSI* myocardial salvage index

Correlations between baseline variables (SS, CK, initial TIMI flow, GRACE score, CRUSADE score) and CMR parameters by Spearman correlation analysis are presented in Table 3. Among them, SS (r = 0.292, *p* < 0.001) and CK (r = 0.328, *p* < 0.001) showed relative stronger correlation with IS (%LV) than other variables. Also, SS (r = -0.314, *p* < 0.001) and initial TIMI flow (r = 0.216, *p* = 0.011) were more correlated with MSI. Therefore, SS had a positive correlation with IS (%LV) and a negative correlation with MSI (Fig. 4).

**Table 3:** Correlations Among baseline variables and CMR parameters

Parameters	IS (%LV, n = 149)	AAR (%LV, n = 149)	MSI (n = 149)	MVO (%IS, n = 54)	MVO (%LV, n = 54)	MVO (ml, n = 54)	IMH (%LV, n = 32)	IMH (%AAR, n = 32)
SYNTAX score								
r	0.292	0.03	− 0.314	0.14	0.18	0.18	0.07	0.05
*p*-value	< 0.001	0.76	< 0.001	0.32	0.20	0.20	0.69	0.80
CK								
r	0.328	0.280	− 0.089	− 0.047	0.034	0.062	− 0.265	− 0.272
*p*-value	< 0.001	0.001	0.306	0.734	0.806	0.657	0.143	0.132
Initial TIMI flow								
r	− 0.148	0.048	0.216	− 0.303	− 0.230	− 0.268	− 0.401	− 0.414
*p*-value	0.071	0.567	0.011	0.026	0.094	0.050	0.023	0.019
GRACE score								
r	0.162	0.080	− 0.056	− 0.141	− 0.011	− 0.036	0.06	0.074
*p*-value	0.079	0.397	0.557	0.329	0.937	0.804	0.759	0.704
CRUSADE score								
r	0.05	0.065	0.011	0.048	0.147	0.065	0.195	0.188
*p*-value	0.591	0.487	0.910	0.741	0.308	0.655	0.310	0.328

*CMR* cardiac magnetic resonance imaging; *IMH* intramyocardial hemorrhage; *MVO* microvascular obstruction; *IS* infarct size; *AAR* area at risk; *MSI* myocardial salvage index

### Univariable and multivariable analysis

**Fig. 5:**
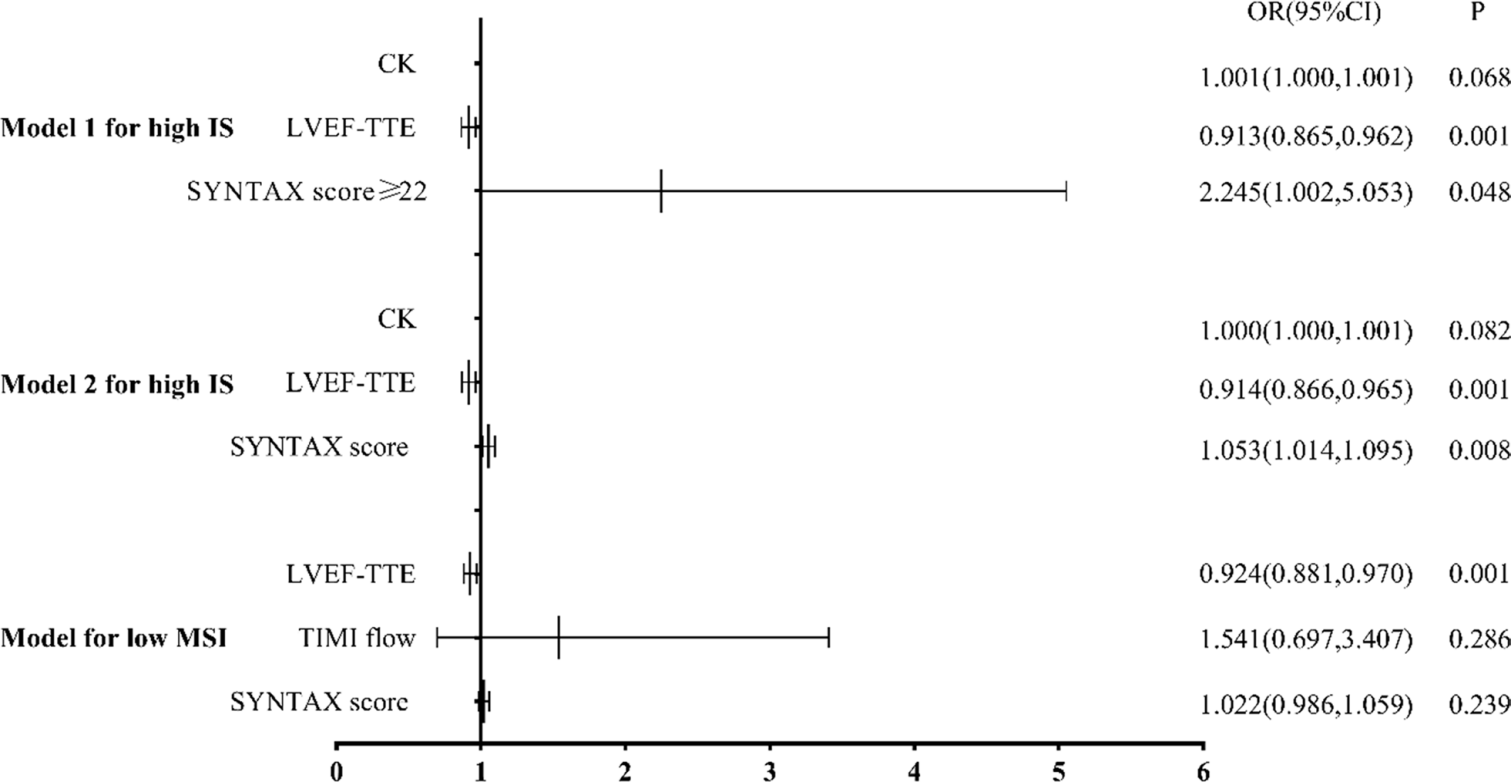
Multivariable logistic regression model for high IS and low MSI. Model1, Model2, Model for low MSI: adjusted for initial heart rate, pro-BNP, CKMB, LVEDD-TTE, IRA = LAD. *OR* odd ratio; *CI* confidence interval; *IS* infarct size; *LVEF* left ventricular ejection fraction; *TTE* transthoracic echocardiography; *MSI* myocardial salvage index; *TIMI* thrombolysis in myocardial infarction

Univariable analysis revealed that nine variables (all *p* < 0.05) were significantly associated with an increased risk of high IS (≥ mean 35.43), including initial heart rate, pro-BNP, CK, CKMB, LVEF-TTE, left ventricular end-diastolic diameter (LVEDD), infarct-related artery LAD, SS > 22 (as a categorical variable) and SS (as a continuous variable); LVEF-TTE, baseline TIMI flow 0/1 and SS (not as a categorical variable) were significant predictors for risk of low MSI (≤ median 28.01) (Table 4). After adjustment for other potential confounders including initial heart rate, pro-BNP, CK-MB, LVEDD-TTE, infarct-related artery LAD, multivariable analysis showed that LVEF-TTE (OR = 0.913, 95%CI [0.865–0.962], *p* = 0.001) and SS > 22 (OR = 2.245, 95%CI [1.002–5.053], *p* = 0.048) were significant for high IS in model 1; in model 2, LVEF-TTE (OR = 0.914, 95%CI [0.866–0.965], *p* = 0.001) and SS (as continuous variable) (OR = 1.053, 95%CI [1.014–1.095], *p* = 0.008) were also the independent predictors of high IS; for low MSI in model 3, only LVEF-TTE (OR = 0.924, 95%CI [0.881–0.970], *p* = 0.001) was the independent predictor. Among the three models, the SS had higher odd ratio than LVEF-TTE (Fig. 5).

**Table 4:** Univariable logistic regression model for high IS and low MSI

Predictors	Univariable model		
	Odds ratio	95% confidence index	*p*-value
High IS (≥ mean 35.43)			
Initial heart rate	1.027	1.001–1.055	0.043
Pro-BNP	1.0	1.0–1.001	0.037
CK	1.001	1.0–1.001	0.010
CKMB	1.005	1.001–1.010	0.020
LVEF-TTE (%)	0.892	0.847–0.939	< 0.001
LVEDD-TTE (mm)	1.062	1.001–1.127	0.045
IRA = LAD (ref. other arteries)	2.214	1.131–4.334	0.020
SYNTAX score			
As continuous variable	1.059	1.024–1.095	0.001
> 22	2.500	1.245–5.019	0.010
Low MSI (≤ median 28.01)			
LVEF-TTE (%)	0.918	0.875–0.962	< 0.001
Baseline TIMI flow 0/1 (ref. TIMI flow 2/3)	2.119	1.049–4.280	0.036
SYNTAX score			
As continuous variable	1.039	1.006–1.073	0.020
> 22	1.561	0.773–3.152	0.214

*IS* infarct size; *IRA* infarct-related artery; *LAD* left anterior descending artery; *MSI* myocardial salvage index; *TIMI* thrombolysis in myocardial infarction

### SYNTAX score in predicting myocardial injury and salvage

**Fig. 6:**
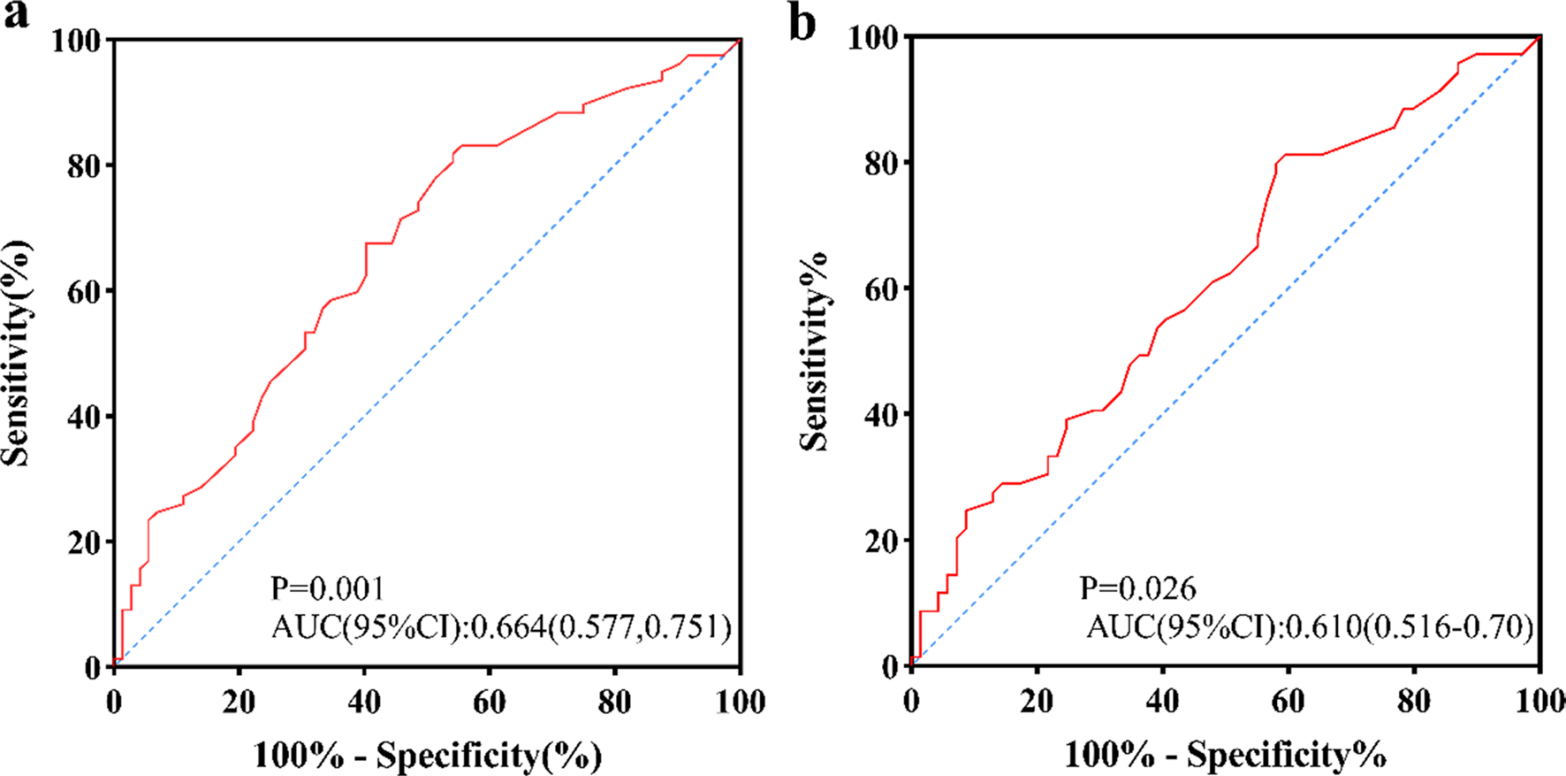
Receiver operating characteristic (ROC) curves for SS to predict high IS (≥ mean 35.43) (sensitivity of 81.8%, specificity of 45.8%) (**a**) and low MSI (≤ median 28.01) (sensitivity of 81.2%, specificity of 40.6%) (**b**). *IS* infarct size; *MSI* myocardial salvage index

The ROC analysis results of SS predicting high IS (≥ mean 35.43) and low MSI (≤ median 28.01) are shown in Fig. 6, with AUC of 0.664 (0.577–0.751, *p* = 0.001), 0.610 (0.516–0.700, *p* = 0.026), respectively. At the optimal cutoff point of SS (10.8 for high IS, 10.3 for low MSI), the sensitivity and specificity were similar for high IS diagnosis (81.8%, 45.8%) and for low MSI (81.2%, 40.6%).

## Discussion

Although the SYNTAX score was shown to positively correlate with postprocedural myocardial injury in patients after elective coronary artery intervention, quality evidence about the association of SS with myocardial salvage in STEMI patients is still needed. This study evaluated the prognostic value of SS for myocardial injury and salvage assessed by CMR after primary PCI in STEMI patients. To the best of our knowledge, the present study is the first report on the relationship between SS and myocardial injury in STEMI patients undergoing primary PCI within 12 h from symptom onset. We identified three major findings: (1) a significant positive correlation between SS and IS and a negative correlation between SS and MSI. (2) compared to the low SS group, the mediate-high group had lower LVEF as assessed by CMR and higher LVESV, but LVEDV was comparable between groups. (3) according to the univariable and multivariable logistic models, SS (both categorical and continuous variables) was the independent predictor of high IS after adjustment for confounders.

In the CvLPRIT study [27], patients treated with a staged complete revascularization (CR) had higher SS (18.3 [15–26] vs. 16 [12–21.5], *p* = 0.021) than those treated with immediate CR. Interestingly, staged approach patients also had larger IS (%LV) (19.1 [10.2–37.1] vs. 11.6 [6.8–17.6], *p* = 0.006) and lower MSI (35.1 [5.9–66.4] vs. 61.7 [37.4–75.5], *p* = 0.008) compared with immediate CR. However, no direct relationship among them was found. In several studies [19, 20, 28, 29], in patients undergone CABG or PCI, SS was not only positively related to peri-procedural myocardial injury (deprived by elevated cardiac troponin and CKMB 6 h after operation) but also an independent predictor of it. The increase in the release of cardiac biomarkers after selective PCI was significantly associated with the extent of atherosclerosis identified by the SS [30]. These findings were consistent with those of our study. Furthermore, in our study, no significant relationship between SS and AAR was found. Thus, the negative correlation between SS and MSI might be interpreted by the positive correlation between SS and IS.

In our study, the LVEF-TTE in the low SS group was comparable with that in the mediate-high SS group (57.63 ± 8.87 vs. 54.77 ± 9.55, *p* = 0.070), however, LVEF-CMR in the low SS group was significantly higher than that in the mediate-high group (52.86 ± 13.45 vs. 45.65 ± 12.01, *p* = 0.001). In addition, among our study population, LVEF-CMR was lower than LVEF-TTE, in line with findings reported by another recent multicenter study [31]. Compared with LVEF-TTE, LVEF-CMR significantly improved MACE prediction in patients with echocardiography-LVEF < 50% and had better prognostic meaning.

The SYNTAX score is not only an angiographic tool used to grade the complexity of coronary artery diseases and guide selecting the proper revascularization strategy [32], but also an important score to predict cardiac mortality [33] and major bleeding (defined as BARC types 3 or 5) risk after drug-eluting stent implantation, although the predictive value was lower than CRUSADE score [34]. Because in-hospital hemoglobin drop is common among patients with acute coronary syndrome [35], the predictive value of syntax score might be better in patients who met the well validated high bleeding risk criteria proposed by the academic research consortium [36]. Further research was needed to validate the hypothesis. Thus, in additional to our main findings, SYNTAX score, like PRAISE model [37], should be used as a multifunctional tool for clinical decision-making.

To specify the influence of SS on myocardial injury after STEMI could have important clinical implications. In China, CMR is available only in large hospitals. Therefore, risk stratification for STEMI patients is very important. By evaluating the SS, we might find the patients who have a high risk of myocardial injury. Despite numerous failures to date, the prevention and treatments for STEMI patients with a high risk of myocardial injury should focus on future cardiovascular research.

This study has a few limitations. Firstly, due to the retrospective nature of the study, selection bias might be present. The proportion of patients within a specific SS group (≤ 22, n = 96; 22 < & ≤ 32, n = 36; > 32, n = 17) in our population was too small to allow conclusions in all three groups. Consequently, tests of interaction were underpowered, especially when adjusted for covariables. Secondly, although all patients were enrolled consecutively, part of them declined CMR for personal reasons or unstable clinical station, for example, severe heart failure; on the other hand, only patients presented in hospital undergone primary PCI within 12 h from symptom onset were included in our study population. Thus, we should be very cautious about interpreting the conclusions in specific patients. Thirdly, considering the “shrinkage” of the infarcted region after the acute infarction phase [2], measurement by CMR may lead to IS overestimation in our study. And finally, in this single-center study, the scoring assessment was performed by highly experienced professionals, and a multi-center fully blinded study might be needed to evaluate further the predictive value of SS in everyday clinical practice.

## Conclusions

In STEMI patients who had undergone primary PCI, SYNTAX score positively correlated with infarct size and negatively with myocardial salvage. SYNTAX score had an independent predictive value of the myocardial injury.

## Data Availability

The data set supporting the results of this article are included within the article. The datasets used and/or analyzed during the current study are available from the corresponding author on reasonable request.

## Abbreviations

SS: SYNTAX score
STEMI: ST-segment elevation myocardial infarction
PCI: Percutaneous coronary intervention
IS: Infarct size
MSI: Myocardial salvage index
MI: Myocardial infarction
MVO: Microvascular obstruction
HF: Heart failure
MACE: Major cardiovascular adverse events
IMH: Intramyocardial hemorrhage
CMR: Cardiac magnetic resonance
PCI: Percutaneous coronary intervention
CAD: Coronary artery disease
ECG: Electrocardiography
SSFP: Steady-state free precession
LV: Left ventricular
AAR: Area at risk
LGE: Late gadolinium enhancement
SD: Standard deviations
ROC: Receiver operating characteristic
TTE: Transthoracic echocardiography
LVEDD: Left ventricular end-diastolic diameter
LAD: Left anterior descending artery
